# Differences in tumour characteristics of Hepatocellular Carcinoma between patients with and without Cirrhosis: A population-based study

**DOI:** 10.7150/jca.46927

**Published:** 2020-08-03

**Authors:** Bing Yan, Dou-Sheng Bai, Jian-Jun Qian, Chi Zhang, Sheng-Jie Jin, Xuehao Wang, Guo-Qing Jiang

**Affiliations:** 1 Department of Hepatobiliary Surgery, Clinical Medical College, Yangzhou University, Yangzhou 225001, China.; 2 Department of Hepatobiliary Surgery, The Second Clinical College, Dalian Medical University, Dalian 116044, China.; 3 Key Laboratory of Living Donor Liver Transplantation, Ministry of Public Health; Department of Liver Transplantation Center, The First Affiliated Hospital of Nanjing Medical University, Nanjing 210029, China.

**Keywords:** Cirrhosis, hepatocellular carcinoma, metastasis, pathological grade, tumour size

## Abstract

**Background:** Liver cirrhosis is a major risk factor for hepatocellular carcinoma (HCC). However, 10%-20% of patients with HCC do not have cirrhosis. The aim of this study was to explore the potential differences in tumour characteristics of HCC between patients with and without cirrhosis.

**Methods:** In this study, we identified total 10,849 patients with HCC diagnosed between 2010 and 2016, from the SEER database. The degree of fibrosis was categorized as “no cirrhosis” (Ishak score 0-4) or “cirrhosis” (Ishak score 5-6). Among all patients with HCC, patients with no cirrhosis and with cirrhosis accounted for 1800 (16.6%) and 9049 (83.4%), respectively.

**Results:** Significant negative correlations were observed between no cirrhosis/cirrhosis and pathological grade (r =-0.074, P <0.001), tumour size (r =-0.186, P <0.001), N stage (r =-0.024, P =0.025), M stage (r =-0.036, P <0.001), liver metastasis (r =-0.024, P =0.014), and lung metastasis (r =-0.027, P =0.006). Logistic multivariate regression analysis showed that, compared with cirrhosis, no cirrhosis is an independent risk predictor of pathological grade [odds ratio (OR), 0.685; 95% confidence interval (CI), 0.571-0.822; P < 0.001], tumour size (OR, 0.392; 95% CI, 0.351-0.437; P < 0.001), N stage (OR, 0.704; 95% CI, 0.561-0.883; P < 0.001), and M stage (OR, 0.671; 95% CI, 0.561-0.804; P < 0.001).

**Conclusions:** Compared with cirrhosis, no cirrhosis is significantly associated with worse pathological grade, larger tumour size, and more lymph node and distant metastases. Patients without cirrhosis that are otherwise neglected in HCC clinical practice require intensive focus in future studies.

## Introduction

Hepatocellular carcinoma (HCC) is the seventh most prevalent cancer worldwide and the fourth leading cause of cancer-related death 1. Liver cirrhosis induced by any aetiology is a major risk factor for HCC and has been associated with infection by hepatitis B virus or hepatitis C virus, alcoholic liver disease, and non-alcoholic fatty liver disease. HCC occurs in approximately 80%-90% of patients with liver cirrhosis 2,3. In other words, about 10%-20% of patients with HCC do not have cirrhosis. However, differences in the tumour characteristics of HCC between patients with and without cirrhosis are still unclear.

In this study, we examined the potential differences in tumour characteristics of HCC between patients with and without cirrhosis using a population-based data from the cancer registry of the Surveillance, Epidemiology, and End Results (SEER) program 4. To the best of our knowledge, the present study is the first report that explores the differences in tumour characteristics of HCC between patients with and without cirrhosis.

## Materials and Methods

### Data Source

The study cohort was assembled using data associated with HCC from the SEER program (from 2010 through 2016). The SEER database is maintained by the US National Cancer Institute and provides information on cancer incidence and survival 4. Initially, 54,238 patients with liver cancer were identified using Site Code C220 in the SEER database. We collected demographic data included sex, age, race, and marital status. Clinical characteristics included year of diagnosis; pathological grade; tumour, node, metastasis (TNM)-7 stage; bone, brain, liver, lung, distant lymph node, and other metastasis; tumour size; alpha fetoprotein (AFP) level; fibrosis score; radiation; chemotherapy; and surgery. Surgery included none, local tumour destruction (photodynamic therapy, electrocautery, fulguration, cryosurgery, laser, percutaneous ethanol injection, Heat-Radio-Frequency ablation, ultrasound, and acetic acid), surgical resection, and liver transplantation. The SEER database classifies fibrosis according to scores defined by the American Joint Committee on Cancer (AJCC) that range from 0 to 4 (undetectable to moderate fibrosis), designated “F0”, and 5 to 6 (incomplete to complete cirrhosis), designated “F1” 5. In this study, we designated “F0” and “F1” as “no cirrhosis” and “cirrhosis”, respectively.

### Patient Selection

**Figure 1:**
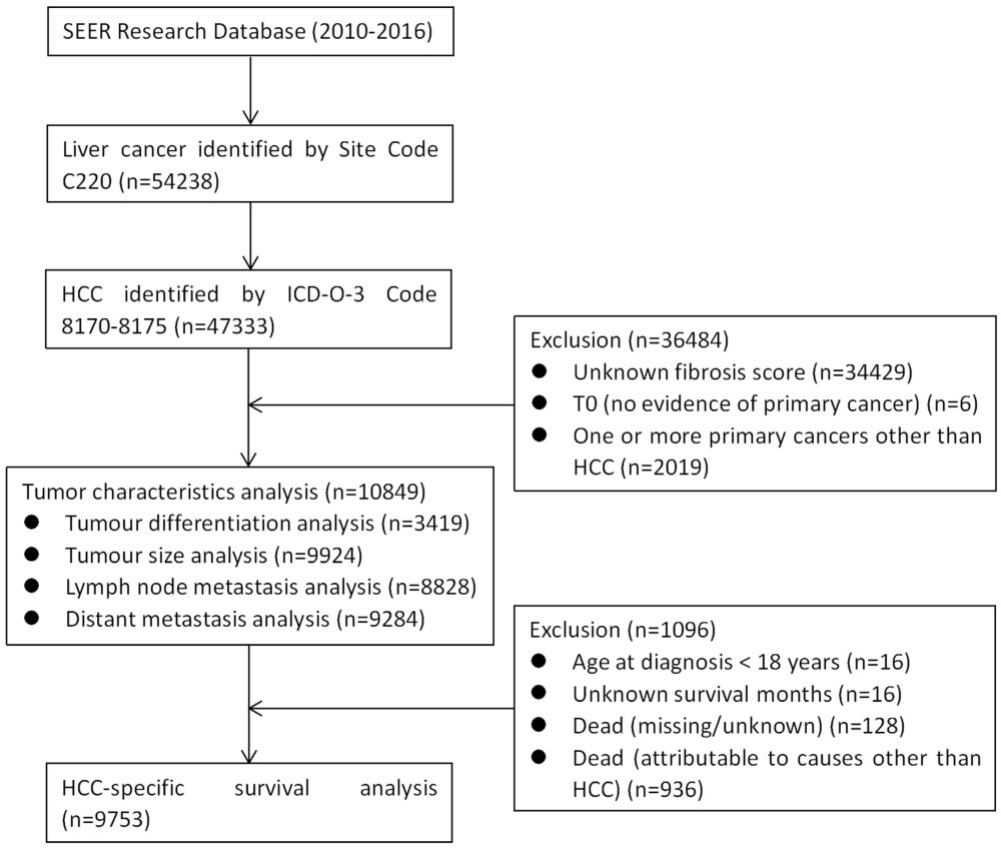
** CONSORT diagram.** SEER: the Surveillance, Epidemiology, and End Results; HCC: hepatocellular carcinoma.

The patient group was reduced to 47,333 patients by inclusion criteria of selecting patients with histologic type HCC, according to the International Classification of Diseases for Oncology, 3rd Edition (ICD-O-3) (codes 8170, 8171, 8172, 8173, 8174 or 8175). We excluded patients with unknown fibrosis score, T0 (no evidence of primary cancer), or one or more primary cancers other than HCC. Thus, as shown in Figure 1, a total of 10,849 patients were included in the analysis of tumour characteristics. Tumour pathological grade, tumour size, N stage, and M stage data were available for 3419, 9924, 8828, and 9284 of these patients, respectively (**Figure 1**). The patient group was further reduced to 9753 patients after meeting the inclusion criteria that including patients those with age ≥ 18 years at diagnosis, definite survival months, or death attributable to HCC. We then performed HCC-specific survival (HCSS) analysis among the remaining patient group.

### Statistical Analyses

Statistical evaluation was conducted using IBM SPSS 22.0 (IBM Corp., Armonk, NY, USA). P values < 0.05 were considered statistically significant. Variables with P < 0.05 in univariate analysis were included in the final multivariate model.HCSS was derived from the dates of diagnosis of HCC and HCC cause-specific death. TNM-7 stages were assigned according to the criteria described in the AJCC Cancer Staging Manual (7th Edition) 6.

The χ^2^ test was used to compare characteristics between the patient groups with and without cirrhosis. Logistic multivariate regression was used to ascertain the different influences of cirrhosis and no cirrhosis on pathological grade, tumour size, N stage (lymph node metastasis), and M stage (distant metastasis). Univariate and multivariable Cox regression analyses were conducted to evaluate the effect of no cirrhosis/cirrhosis on HCSS.

## Results

### Patient Baseline Characteristics

Among the total 10,849 patients, 9049 (83.4%) had cirrhosis and 1800 (16.6%) patients did not have cirrhosis. As shown in **Table 1**, a comparative analysis of baseline demographics and tumour characteristics of groups with and without cirrhosis revealed that the no-cirrhosis group had higher proportions of older (age ≥ 60 years) and married patients, N1 stage, M1 stage, liver metastasis, lung metastasis, and negative AFP (all P < 0.05).

### Association between no cirrhosis/cirrhosis and pathological grade

There were a total 3419 patients with precise pathological grade information. A significant negative correlation was observed between no cirrhosis/cirrhosis and pathological grade (r = -0.074, P < 0.001). The no-cirrhosis group had more poorly differentiated/anaplastic tumours than the cirrhosis group (24.4% vs. 18.4%, P < 0.001). As shown in **Table 2**, univariate analysis of seven variables was conducted between well/moderately differentiated and poorly differentiated/anaplastic tumours. Significant variables between the two groups included race, AFP level, and no cirrhosis/cirrhosis. These statistically significant variables were regarded as independent variables, and pathological grade was regarded as the dependent variable. Logistic regression analysis revealed that, compared with no cirrhosis, cirrhosis was an independent and protective predictor of pathological grade [odds ratio (OR), 0.685; 95% confidence interval (CI), 0.571-0.822; P < 0.001].

### Association between no cirrhosis/cirrhosis and tumour size

Analysis of 9902 patients with information of precise tumour size showed a significant negative correlation between no cirrhosis/cirrhosis and tumour size (r = -0.186, P < 0.001), and tumour size in the no-cirrhosis group was significantly larger than in the cirrhosis group (68.0 ± 54.3 mm vs. 46.3 ± 40.3 mm, P < 0.001).

There were a total 9924 patients with rough tumour size information. More tumours with size ≥ 5 cm were found in the group without cirrhosis than in the cirrhosis group (50.9% vs. 29.5%, P < 0.001). As shown in **Table 3**, univariate analysis of seven variables was conducted between tumour size < 5 cm and ≥ 5 cm; significant variables between the two groups included sex, age, race, AFP level, and no cirrhosis/cirrhosis. The statistically significant variables were regarded as independent variables, and tumour size was regarded as the dependent variable. Logistic regression analysis revealed that, compared with no cirrhosis, cirrhosis was an independent and protective predictor of tumour size (OR, 0.392; 95% CI, 0.351-0.437; P < 0.001).

### Association between no cirrhosis/cirrhosis and N stage

There were a total 8828 patients with original N stage information. A significant negative correlation was observed between no cirrhosis/cirrhosis and N stage (r = -0.025, P < 0.05). More N1 stage tumours occurred in the group without cirrhosis than in the cirrhosis group (7.2% vs. 5.6%, P < 0.001). As shown in **Table 4**, univariate analysis of seven variables between N0 stage and N1 stage tumours revealed that significant variables between the two groups included sex, race, marital status, AFP level, and no cirrhosis/cirrhosis. Statistically significant variables were regarded as independent variables, and N stage was regarded as the dependent variable. Logistic regression analysis revealed that, compared with no cirrhosis, cirrhosis was an independent and protective predictor of N stage (OR, 0.704; 95% CI, 0.561-0.883; P < 0.001).

### Association between no cirrhosis/cirrhosis and M stage

There were a total 8828 patients with original M stage information. A significant negative correlation was observed between no cirrhosis/cirrhosis and M stage (r = -0.036, P < 0.001). More M1 stage tumours were observed in patients without cirrhosis than in those with cirrhosis (11.8% vs. 8.9%, P < 0.001). As shown in **Table 5**, univariate analysis of seven variables between M0 stage and M1 stage tumours identified that significant variables between the two groups included sex, age, ethnicity, marital status, AFP level, and no cirrhosis/cirrhosis. Statistically significant variables were regarded as independent variables, and M stage was regarded as the dependent variable. Logistic regression analysis revealed that, compared with no cirrhosis, cirrhosis was an independent and protective predictor of M stage (OR, 0.671; 95% CI, 0.561-0.804; P < 0.001).

We identified 10,586 patients with liver metastasis information and found that patients without cirrhosis had a higher proportion of liver metastasis than those with cirrhosis: 16/1760 (0.9%) versus 39/8771 (0.4%), P = 0.014. Furthermore, the analysis of 10,571 patients with information on lung metastasis showed that the group without cirrhosis had a higher proportion of lung metastasis than the cirrhosis group: 82/1695 (4.6%) versus 289/8505 (3.3%), P = 0.006. There were significant negative correlations between no cirrhosis/cirrhosis and liver metastasis (r = -0.024, P = 0.014), and lung metastasis (r = -0.027, P = 0.006).

### Association between no cirrhosis/cirrhosis and HCSS

As shown in **Table 6**, univariate Cox proportional hazards analysis involving 9753 patients was conducted to evaluate the association between different clinical variables and HCSS. Sex; race; marital status; year of diagnosis; pathological grade; T, N, and M stages; tumour size; AFP level; radiation; chemotherapy; surgery; and no cirrhosis/cirrhosis were identified as significant predictors for survival. Multivariable Cox regression analysis revealed that, compared with no cirrhosis, cirrhosis was an independent and risk prognostic factor for HCSS (hazard ratio [HR], 1.166; 95% CI, 1.074-1.265; P < 0.001).

## Discussion

It is well known that cirrhosis is a major risk factor for hepatocarcinogenesis. As shown in the present study, among all patients with HCC, the number of patients with cirrhosis was over five times (9049/1800) that of patients without cirrhosis. However, the relationship between no cirrhosis/cirrhosis and tumour characteristics of HCC has not been reported. It has been unclear whether, compared with no cirrhosis; cirrhosis would be associated with larger tumour size or a greater number of less-differentiated tumours, or lymph node or distant metastasis.

The aim of the present study was to better understand the effect of no cirrhosis/cirrhosis on tumour characteristics, particularly on tumour differentiation, tumour size, lymph node metastasis, and distant metastasis. Improved knowledge of the effect of no cirrhosis/cirrhosis on tumour characteristics would help to properly classify patients with advanced stages of disease and may serve as a reference for personalized, precise treatment. It would seem logical that compared with no cirrhosis, cirrhosis would be associated with less differentiated tumours, more advanced TNM stage, and greater tumour size; however, the present study led to the opposite conclusions.

We have tried to use some publicly available databases including cBioPortal, GEO, TCGA, and so on, to validate our main findings and conclusions. However, we found that these publicly available databases concerning gene research are unsuited to the validation.

In order to obtain patients' genetic information, common defects of these publicly available databases is that most of the selected patients had received liver resection for HCC, for example, the data from cBioPortal databases showed 205 (95.3%) patients have underwent liver resection among 215 patients with fibrosis information 7. In fact, it is well known that the majority of HCC patients at diagnosis lost the chance to receive liver resection owing to too big tumor or metastases, these HCC usually have more malignant biological behaviour than those who received liver resection. So, these publicly available databases used a biased inclusion criteria. The rate of patients who were not performed surgical treatment was about 69.6% (7549/10849) in the current study and 65.6% (7726/11783) in a previous study 8. Therefore, these publicly available databases cannot reflect the real world of HCC, and the investigation outcomes from these databases may bring about biased conclusions.

However, we can use the data of the previous studies by the means of reanalysis and logical reasoning, to a certain extent, to validate our main findings and conclusions, as shown in the following discussion 8,9.

The present study revealed that no cirrhosis/cirrhosis had a significant negative correlation with pathological grade. With progression of pathological grade, the proportions of different pathological grades in the no-cirrhosis group ascended dynamically, in comparison with the cirrhosis group, as follows. First, the incidence of well differentiated tumours in the no-cirrhosis group was lower than that in the cirrhosis group (26.5% vs. 32.1%); the incidence of moderately differentiated tumours was similar in both groups (49.1% vs. 49.5%); finally, the incidences of poorly differentiated and undifferentiated tumours in the no-cirrhosis group were all higher than those in the cirrhosis group (23.0% vs. 17.3%, 1.4% vs. 1.1%, respectively). In addition, the present study demonstrated that no cirrhosis is an independent risk predictor for less-differentiated tumours, as compared with cirrhosis. Patients without cirrhosis were about 1.5 (1/0.685) times as likely to have poorly differentiated/anaplastic tumours as those with cirrhosis.

A previous study based on SEER database showed there were no significant differences in pathological grade between patients with and without cirrhosis 9. This findings does not coincides with our present findings, which may due to their specific inclusion criteria that only patients who had received liver resection for HCC was included in the study. In fact, it is well known that the majority of HCC patients at diagnosis lost the chance to receive liver resection owing to too big tumor or metastases, these HCC usually have more malignant biological behaviour than those who received liver resection. The rate of patients who were not performed surgical treatment was about 69.6% (7549/10849) in the current study and 65.6% (7726/11783) in another retrospective SEER study 8. Therefore, that previous study cannot reflect the real world of HCC, and its investigation outcomes may bring about biased conclusions.

Liu et al. reported that cirrhosis was positively correlated with advanced pathological grade (r = 0.19, P < 0.001) 8. It seems that this conclusion was in contrast to ours, which is owing to their false statistical analysis. During the analysis of the relationship of between no cirrhosis/cirrhosis and pathological grade, the majority (62.2%, 7328/11783) of patients with unavailable pathological grade information contributed to distorted outcome. When we deleted the data of that previous study for patients with unknown information of pathological grade and reanalyzed the data, we found, in fact, that cirrhosis was negatively correlated with advanced pathological grade (r = -0.287, P < 0.001), which was consistent with our present findings.

Compared with cirrhosis, a significant positive correlation was observed between no cirrhosis and N1 stage in the present study; in addition, logistic multivariate regression showed no cirrhosis was an independent risk predictor for lymph node metastasis. Our findings showed that patients without cirrhosis were more than 1.4 (1/0.704) times as likely to have lymph node metastasis as those with cirrhosis. Furthermore, compared with cirrhosis, a significant positive correlation was found between no cirrhosis and M1 stage in the present study; in addition, logistic multivariate regression showed no cirrhosis was an independent risk predictor for distant metastasis. Patients without cirrhosis were approximately 1.5 (1/0.671) times as likely to have distant metastasis as those with cirrhosis. The incidences of distant organ metastasis in the brain (0.2%), liver (0.5%), bone (2.6%), and lung (3.5%) were gradually elevated. The lung was the most common site of metastasis and the brain was the least common site. There were significant negative correlations between no cirrhosis/cirrhosis and liver metastasis, and lung metastasis. Among all distant metastases, no cirrhosis was a significant risk factor for liver and lung metastasis, as compared with cirrhosis. The data of a previous study showed more TNM-stage IV tumours occurred in the group without cirrhosis than in the cirrhosis group (12.1% vs. 9.2%, P < 0.001) 8. As we all know, TNM-stage IV means tumour is at N1 or M1 stageS. Therefore, this data basically supported our findings.

A significant negative correlation was observed between no cirrhosis/cirrhosis and tumour size in the present study. Tumour size in the no-cirrhosis group was about 1.5 (68.0 mm/46.3 mm) times that of the cirrhosis group; in addition, logistic multivariate regression showed no cirrhosis was an independent risk predictor for larger tumours. This finding was supported by that of a previous study they demonstrated that, compared with cirrhosis, no cirrhosis was negatively related to smaller tumour (r = -0.16, P < 0.001) 8. Another study also revealed that no cirrhosis is significantly associated with larger tumour, which is consistent with our conclusion 9.

Despite our finding that no cirrhosis is associated with worse tumour characteristics, the current study showed that cirrhosis was independently associated with poorer HCSS. The latter finding has been supported in many studies 9,10, which have reported that cirrhosis is an independently significant predictor of poor survival following surgical resection. Background cirrhosis may be the most important factor explaining why HCC patients with cirrhosis have relatively better tumour characteristics but shorter survival than those without cirrhosis. Especially in the case of medical treatment, HCC patients with cirrhosis have a higher risk of developing hepatic dysfunction and even death than those without cirrhosis.

Our data showed that the opportunity for surgical resection in the no-cirrhosis group was 5.8 (34.2%/5.9%) times that in the cirrhosis group, and adjuvant therapy including radiation and chemotherapy was available, to improve HCSS. Therefore, among patients with HCC with no opportunity for surgery, well-selected patients may obtain survival benefit from sufficient administration of radiation or chemotherapy.

The present study has several limitations. First, the SEER HCC database lacks data regarding the aetiologies of patient fibrosis, comorbidities, and recurrence; these variables may affect tumour characteristics and survival. Second, some data for radiation or chemotherapy were denoted “No/Unknown”; this is somewhat unclear and means that in our analysis, we did not have a patient group that definitely did not receive either therapy. Finally, only two categories of fibrosis (“F0” and “F1”) are recorded in the database; if information for an original fibrosis score could be obtained, we could conduct further analyses.

In conclusion, this population-based study demonstrated that no cirrhosis is an independent risk predictor for larger tumour size, worse pathological grade, and lymph node and distant metastases, in comparison with cirrhosis. Patients without cirrhosis, who are otherwise neglected in HCC clinical practice, require additional investigation regarding the specific molecular mechanisms by which they are more prone to worse tumour characteristics. Physician should pay greater attention to patients with HCC who do not have cirrhosis as they may benefit from sufficient and precise therapy.

## Figures and Tables

**Table 1:** Baseline demographic and tumour characteristics of patients with and without cirrhosis

Variables	n=10849	Total	Fibrosis, No. (%)		*P*	No unknown^†^	Fibrosis, No. (%)		*P*
			No cirrhosis	Cirrhosis			No cirrhosis	Cirrhosis	
Sex	Male	8458 (78.0)	1383 (76.8)	7075 (78.2)	0.206	8458 (78.0)	1383 (76.8)	7075 (78.2)	0.206
	Female	2391 (22.0)	417 (23.2)	1974 (21.8)		2391 (22.0)	417 (23.2)	1974 (21.8)	
									
Age	<60	4351 (40.1)	628 (34.9)	3723 (41.1)	< 0.001	4351 (40.1)	628 (34.9)	3723 (41.1)	< 0.001
	≥60	6498 (59.9)	1172 (65.1)	5326 (58.9)		6498 (59.9)	1172 (65.1)	5326 (58.9)	
Race	White	7469 (68.8)	1034 (57.4)	6435 (71.1)	< 0.001	7469 (69.3)	1034 (57.9)	6435 (71.6)	< 0.001
	Black^‡^	1369 (12.6)	267 (14.8)	1102 (12.2)		1369 (12.7)	267 (14.9)	1102 (12.3)	
	Other*	1942 (17.9)	486 (27.0)	1456 (16.1)		1942 (18.0)	486 (27.2)	1456 (16.2)	
	Unknown	69 (0.6)	13 (0.7)	56 (0.6)		-			
Marital Status	Married	5326 (49.1)	973 (54.1)	4353 (48.1)	< 0.001	5326 (51.0)	973 (56.2)	4353 (49.9)	< 0.001
	Non-married^#^	5122 (47.2)	758 (42.1)	4364 (48.2)		5122 (49.0)	758 (43.8)	4364 (50.1)	
	Unknown	401 (3.7)	69 (3.8)	332 (3.7)		-			
Year of diagnosis	2010-2011	2803 (25.8)	434 (24.1)	2369 (26.2)	0.003	2803 (25.8)	434 (24.1)	2369 (26.2)	0.003
	2012-2013	3153 (29.1)	488 (27.1)	2665 (29.5)		3153 (29.1)	488 (27.1)	2665 (29.5)	
	2014-2016	4893 (45.1)	878 (48.8)	4015 (44.4)		4893 (45.1)	878 (48.8)	4015 (44.4)	
Pathological grade	Well differentiated	1039 (9.6)	274 (15.2)	765 (8.5)	< 0.001	1039 (30.4)	274 (26.5)	765 (32.1)	< 0.001
	Moderately differentiated	1688 (15.6)	507 (28.2)	1181 (13.1)		1688 (49.4)	507 (49.1)	1181 (49.5)	
	Poorly differentiated	651 (6.0)	238 (13.2)	413 (4.6)		651 (19.0)	238 (23.0)	413 (17.3)	
	Undifferentiated	41 (0.4)	14 (0.8)	27 (0.3)		41 (1.2)	14 (1.4)	27 (1.1)	
	Unknown	7430 (68.5)	767 (42.6)	6663 (73.6)		-			
T	T1	4009 (37.0)	719 (39.9)	3290 (36.4)	< 0.001	4009 (45.4)	719 (50.0)	3290 (44.5)	< 0.001
	T2	2533 (23.3)	287 (15.9)	2246 (24.8)		2533 (28.7)	287 (19.9)	2246 (30.4)	
	T3	2028 (18.7)	364 (20.2)	1664 (18.4)		2028 (23.0)	364 (25.3)	1664 (22.5)	
	T4	265 (2.4)	69 (3.8)	196 (2.2)		265 (3.0)	69 (4.8)	196 (2.7)	
	Unknown	2014 (18.6)	361 (20.1)	1653 (18.3)		-			
N	N0	8309 (76.6)	1338 (74.3)	6971 (77.0)	0.025	8309 (94.1)	1338 (92.9)	6971 (94.4)	0.025
	N1	519 (4.8)	103 (5.7)	416 (4.6)		519 (5.9)	103 (7.1)	416 (5.6)	
	Unknown	2021 (18.6)	359 (19.9)	1662 (18.4)		-			
M	M0	8413 (77.5)	1313 (72.9)	7100 (78.5)	< 0.001	8413 (90.6)	1313 (88.2)	7100 (91.1)	< 0.001
	M1	871 (8.0)	175 (9.7)	696 (7.7)		871 (9.4)	175 (11.8)	696 (8.9)	
	Unknown	1565 (14.4)	312 (17.3)	1253 (13.8)		-			
Bone metastasis	Yes	279 (2.6)	49 (2.7)	230 (2.5)	0.003	279 (2.6)	49 (2.8)	230 (2.6)	0.725
	No	10308 (95.0)	1728 (96.0)	8580 (94.8)		10308 (97.4)	1728 (97.2)	8580 (97.4)	
	Unknown	262 (2.4)	23 (1.3)	239 (2.6)		-			
Brain metastasis	Yes	20 (0.2)	1 (0.1)	19 (0.2)	0.003	20 (0.2)	1 (0.1)	19 (0.2)	0.232
	No	10557 (97.3)	1773 (98.5)	8784 (97.1)		10557 (99.8)	1773 (99.9)	8784 (99.8)	
	Unknown	272 (2.5)	26 (1.4)	246 (2.7)		-			
Liver metastasis	Yes	55 (0.5)	16 (0.9)	39 (0.4)	< 0.001	55 (0.5)	16 (0.9)	39 (0.4)	0.014
	No	10531 (97.1)	1760 (97.8)	8771 (96.9)		10531 (99.5)	1760 (99.1)	8771 (99.6)	
	Unknown	263 (2.4)	24 (1.3)	239 (2.6)		-			
Lung metastasis	Yes	371 (3.4)	82 (4.6)	289 (3.2)	< 0.001	371 (3.5)	82 (4.6)	289 (3.3)	0.006
	No	10200 (94.0)	1695 (94.2)	8505 (94.0)		10200 (96.5)	1695 (95.4)	8505 (96.7)	
	Unknown	278 (2.6)	23 (1.3)	255 (2.8)		-			
Distant lymph node metastasis	Yes	27 (0.2)	6 (0.3)	21 (0.2)	< 0.001	27 (1.8)	6 (2.0)	21 (1.7)	0.780
	No	1496 (13.8)	300 (16.7)	1196 (13.2)		1496 (98.2)	300 (98.0)	1196 (98.3)	
	Unknown	9326 (86.0)	1494 (83.0)	7832 (86.6)		-			
Other metastasis	Yes	36 (0.3)	10 (0.6)	26 (0.3)	< 0.001	36 (2.4)	10 (3.3)	26 (2.1)	0.246
	No	1485 (13.7)	296 (16.4)	1189 (13.1)		1485 (97.6)	296 (96.7)	1189 (97.9)	
	Unknown	9328 (86.0)	1494 (83.0)	7834 (86.6)		-			
Tumor Size	≤3 cm	3991 (36.8)	463 (25.7)	3528 (39.0)	< 0.001	3991 (40.2)	463 (27.5)	3528 (42.8)	< 0.001
	3-5 cm	2469 (22.8)	344 (19.1)	2125 (23.5)		2469 (24.9)	344 (20.4)	2125 (25.8)	
	≥5 cm	3464 (31.9)	879 (48.8)	2585 (28.6)		3464 (34.9)	879 (52.1)	2585 (31.4)	
	Unknown	925 (8.5)	114 (6.3)	811 (9.0)		-			
AFP	Negative	2785 (25.7)	543 (30.2)	2242 (24.8)	< 0.001	2785 (29.1)	543 (34.7)	2242 (28.0)	< 0.001
	Positive	6801 (62.7)	1023 (56.8)	5778 (63.9)		6801 (70.9)	1023 (65.3)	5778 (72.0)	
	Borderline	17 (0.2)	5 (0.3)	12 (0.1)		-			
	Unknown	1246 (11.5)	229 (12.7)	1017 (11.2)		-			
Radiation	Yes	924 (8.5)	169 (9.4)	755 (8.3)	0.147	-			
	None/unknown	9925 (91.5)	1631 (90.6)	8294 (91.7)		-			
Chemotherapy	Yes	5135 (47.3)	688 (38.2)	4447 (49.1)	< 0.001	-			
	None/unknown	5714 (52.7)	1112 (61.8)	4602 (50.9)		-			
Surgery	None	7549 (69.6)	926 (51.4)	6623 (73.2)	< 0.001	7549 (69.7)	926 (51.6)	6623 (73.3)	< 0.001
	Tumor Destruction	1363 (12.6)	175 (9.7)	1188 (13.1)		1363 (12.6)	175 (9.7)	1188 (13.2)	
	Surgical Resection	1150 (10.6)	615 (34.2)	535 (5.9)		1150 (10.6)	615 (34.2)	535 (5.9)	
	Liver Transplantation	767 (7.1)	80 (4.4)	687 (7.6)		767 (7.1)	80 (4.5)	687 (7.6)	
	Unknown	20 (0.2)	4 (0.2)	16 (0.2)		-			

^†^, Not including unknown variables; ^‡^, Black or African American; *, Includes American Indian/Alaska native, Asian, and Asian/Pacific Islander; ^#^, Includes widowed, never married, divorced, separated, unmarried, and domestic partner; T: tumour; N: node; M: metastasis; AFP: alpha fetoprotein.

**Table 2:** Evaluation of the influence of no cirrhosis/cirrhosis on tumour pathological grade

Variables	n = 3419	Univariate analysis			Logistic multivariate regression	
		Well/Moderate No. (%)	Poor/Anaplastic No. (%)	*P*	OR (95%CI)	*P*
Sex	Male	2128 (78.0)	520 (75.1)	0.104	-	
	Female	599 (22.0)	172 (24.9)		-	
Age	<60	1073 (39.3)	263 (38.0)	0.518	-	
	≥60	1654 (60.7)	429 (62.0)		-	
Race	White	1842 (67.5)	407 (58.8)	< 0.001	Reference	
	Black^‡^	362 (13.3)	92 (13.3)		1.052 (0.814-1.360)	0.697
	Other*	503 (18.4)	191 (27.6)		1.590 (1.297-1.950)	< 0.001
	Unknown	20 (0.7)	2 (0.3)		0.451 (0.103-1.966)	0.289
Marital Status	Married	1517 (55.6)	377 (54.5)	0.849	-	
	Non-married^#^	1118 (41.0)	292 (42.2)		-	
	Unknown	92 (3.4)	23 (3.3)		-	
Year of diagnosis	2010-2011	779 (28.6)	189 (27.3)	0.174	-	
	2012-2013	780 (28.6)	180 (26.0)		-	
	2014-2016	1168 (42.8)	323 (46.7)		-	
AFP	Negative	879 (32.2)	107 (15.5)	< 0.001	Reference	
	Positive	1488 (54.6)	496 (71.7)		2.834 (2.257-3.559)	< 0.001
	Borderline	3 (0.1)	0 (0.0)		0.000 (0.000-0.000)	0.999
	Unknown	357 (13.1)	89 (12.9)		2.120 (1.556-2.888)	< 0.001
Fibrosis	Non-cirrhosis	781 (28.6)	252 (36.4)	< 0.001	Reference	
	Cirrhosis	1946 (71.4)	440 (63.6)		0.685 (0.571-0.822)	< 0.001

^‡^, Black or African American; *, Includes American Indian/Alaska native, Asian, and Asian/Pacific Islander; ^#^, Includes widowed, never married, divorced, separated, unmarried, and domestic partner; AFP: alpha fetoprotein; OR: odds ratio; CI: confidence interval.

**Table 3:** Evaluation of the influence of no cirrhosis/cirrhosis on tumour size

Variables	n = 9924	Univariate analysis			Logistic multivariate regression	
		<5 cm, No. (%)	≥5 cm, No. (%)	*P*	OR (95%CI)	*P*
Sex	Male	5056 (76.2)	2645 (80.4)	< 0.001	Reference	
	Female	1579 (23.8)	644 (19.6)		0.734 (0.660-0.816)	< 0.001
Age	<60	2700 (40.7)	1266 (38.5)	0.035	Reference	
	≥60	3935 (59.3)	2023 (61.5)		1.104 (1.011-1.206)	0.028
Race	White	4715 (71.1)	2110 (64.2)	< 0.001	Reference	
	Black^‡^	767 (11.6)	471 (14.3)		1.230 (1.081-1.400)	0.002
	Other*	1109 (16.7)	691 (21.0)		1.266 (1.132-1.416)	< 0.001
	Unknown	44 (0.7)	17 (0.5)		0.900 (0.505-1.604)	0.721
Marital Status	Married	3336 (50.3)	1617 (49.2)	0.402	-	
	Non-married^#^	3072 (46.3)	1546 (47.0)		-	
	Unknown	227 (3.4)	126 (3.8)		-	
Year of diagnosis	2010-2011	1740 (26.2)	818 (24.9)	0.103	-	
	2012-2013	1936 (29.2)	931 (28.3)		-	
	2014-2016	2959 (44.6)	1540 (46.8)		-	
AFP	Negative	1987 (29.9)	689 (20.9)	< 0.001	Reference	
	Positive	3918 (59.1)	2299 (69.9)		1.230 (1.081-1.400)	< 0.001
	Borderline	14 (0.2)	2 (0.1)		1.266 (1.132-1.416)	
	Unknown	716 (10.8)	299 (9.1)		0.900 (0.505-1.604)	< 0.001
Fibrosis	Non-cirrhosis	827 (12.5)	859 (26.1)	< 0.001	Reference	
	Cirrhosis	5808 (87.5)	2430 (73.9)		0.392 (0.351-0.437)	< 0.001

^‡^, Black or African American; *, Includes American Indian/Alaska native, Asian, and Asian/Pacific Islander; ^#^, Includes widowed, never married, divorced, separated, unmarried, and domestic partner; AFP: alpha fetoprotein; OR: odds ratio; CI: confidence interval.

**Table 4:** Evaluation of the influence of no cirrhosis/cirrhosis on lymph node metastasis

Variables	n = 8828	Univariate analysis			Logistic multivariate regression	
		N0, No. (%)	N1, No. (%)	*P*	OR (95%CI)	*P*
Sex	Male	6464 (77.8)	440 (84.8)	< 0.001	Reference	
	Female	1845 (22.2)	79 (15.2)		0.615 (0.481-0.787)	< 0.001
Age	<60	3417 (41.1)	219 (42.2)	0.630	-	
	≥60	4892 (58.9)	300 (57.8)		-	
Race	White	5708 (68.7)	371 (71.5)	0.029	Reference	
	Black^‡^	1040 (12.5)	76 (14.6)		0.999 (0.771-1.294)	0.992
	Other*	1517 (18.3)	69 (13.3)		0.713 (0.545-0.933)	0.014
	Unknown	44 (0.5)	3 (0.6)		1.178 (0.361-3.845)	0.786
Marital Status	Married	4161 (50.1)	226 (43.5)	0.008	Reference	
	Non-married^#^	3862 (46.5)	268 (51.6)		1.252 (1.039-1.508)	0.018
	Unknown	286 (3.4)	25 (4.8)		1.605 (1.040-2.476)	0.033
Year of diagnosis	2010-2011	2470 (29.7)	144 (27.7)	0.433	-	
	2012-2013	2794 (33.6)	188 (36.2)		-	
	2014-2016	3045 (36.6)	187 (36.0)		-	
AFP	Negative	2180 (26.2)	75 (14.5)	< 0.001	Reference	
	Positive	5211 (62.7)	398 (76.7)		2.250 (1.747-2.898)	< 0.001
	Borderline	14 (0.2)	0 (0.0)		0.000 (0.000-0.000)	0.999
	Unknown	904 (10.9)	46 (8.9)		1.456 (0.999-2.122)	0.050
Fibrosis	Non-cirrhosis	1338 (16.1)	103 (19.8)	0.025	Reference	
	Cirrhosis	6971 (83.9)	416 (80.2)		0.704 (0.561-0.883)	0.002

^‡^, Black or African American; *, Includes American Indian/Alaska native, Asian, and Asian/Pacific Islander; ^#^, Includes widowed, never married, divorced, separated, unmarried, and domestic partner; AFP: alpha fetoprotein; OR: odds ratio; CI: confidence interval.

**Table 5:** Evaluation of the influence of no cirrhosis/cirrhosis on distant metastasis

Variables	n = 9284	Univariate analysis			Logistic multivariate regression	
		M0, No. (%)	M1, No. (%)	P	OR (95%CI)	P
Sex	Male	6550 (77.9)	732 (84.0)	< 0.001	Reference	
	Female	1863 (22.1)	139 (16.0)		0.651 (0.538-0.789)	< 0.001
Age	<60	3436 (40.8)	412 (47.3)	< 0.001	Reference	
	≥60	4977 (59.2)	459 (52.7)		0.807 (0.700-0.930)	0.003
Race	White	5810 (69.1)	589 (67.6)	0.009	Reference	
	Black‡	1029 (12.2)	138 (15.8)		1.169 (0.957-1.428)	0.127
	Other*	1526 (18.1)	142 (16.3)		0.940 (0.772-1.145)	0.539
	Unknown	48 (0.6)	2 (0.2)		0.459 (0.110-1.907)	0.284
Marital Status	Married	4198 (49.9)	376 (43.2)	< 0.001	Reference	
	Non-married^#^	3898 (46.3)	466 (53.5)		1.311 (1.132-1.519)	< 0.001
	Unknown	317 (3.8)	29 (3.3)		0.987 (0.663-1.470)	0.949
Year of diagnosis	2010-2011	2547 (30.3)	256 (29.4)	0.811	-	
	2012-2013	2858 (34.0)	295 (33.9)		-	
	2014-2016	3008 (35.8)	320 (36.7)		-	
AFP	Negative	2201 (26.2)	111 (12.7)		Reference	
	Positive	5216 (62.0)	668 (76.7)		2.530 (2.054-3.116)	< 0.001
	Borderline	13 (0.2)	1 (0.1)		1.466 (0.189-11.377)	0.714
	Unknown	983 (11.7)	91 (10.4)		1.825 (1.368-2.436)	< 0.001
Fibrosis	Non-cirrhosis	1313 (15.6)	175 (20.1)	< 0.001	Reference	
	Cirrhosis	7100 (84.4)	696 (79.9)		0.671 (0.561-0.804)	< 0.001

^‡^, Black or African American; *, Includes American Indian/Alaska native, Asian, and Asian/Pacific Islander; ^#^, Includes widowed, never married, divorced, separated, unmarried, and domestic partner; AFP: alpha fetoprotein; OR: odds ratio; CI: confidence interval.

**Table 6:** Univariate and multivariate Cox proportional hazards analysis of disease-specific survival

Variables	n = 9753	Univariate analysis		Multivariate analysis	
		HR (95 % CI)	*P* Value	HR (95 % CI)	*P* Value
Sex	Male	Reference	< 0.001	Reference	
	Female	0.812 (0.759-0.869)		0.924 (0.862-0.991)	0.027
Age	<60	Reference	0.016	Reference	
	≥60	1.070 (1.013-1.131)		1.102 (1.041-1.166)	< 0.001
Race	White	Reference		Reference	
	Black^‡^	1.103 (1.018-1.196)	0.016	0.935 (0.862-1.014)	0.104
	Other*	0.786 (0.729-0.848)	< 0.001	0.815 (0.754-0.881)	< 0.001
	Unknown	0.573 (0.373-0.880)	0.011	0.695 (0.452-1.068)	0.097
Marital Status	Married	Reference		Reference	
	Non-married^#^	1.317 (1.246-1.392)	< 0.001	1.034 (0.976-1.094)	0.255
	Unknown	1.204 (1.041-1.392)	0.013	0.949 (0.819-1.099)	0.482
Year of diagnosis	2010-2011	Reference		Reference	
	2012-2013	0.983 (0.920-1.051)	0.614	0.992 (0.928-1.061)	0.815
	2014-2016	0.797 (0.743-0.854)	< 0.001	0.873 (0.811-0.940)	< 0.001
Pathological grade	Well differentiated	Reference		Reference	
	Moderately differentiated	1.071 (0.944-1.215)	0.288	1.153 (1.014-1.311)	0.030
	Poorly differentiated	1.931 (1.672-2.230)	< 0.001	1.660 (1.432-1.924)	< 0.001
	Undifferentiated	2.270 (1.515-3.402)	< 0.001	2.121 (1.409-3.193)	< 0.001
	Unknown	1.893 (1.704-2.102)	< 0.001	1.208 (1.085-1.345)	< 0.001
T	T1	Reference		Reference	
	T2	1.387 (1.288-1.495)	< 0.001	1.485 (1.375-1.605)	< 0.001
	T3	4.076 (3.793-4.381)	< 0.001	1.878 (1.722-2.048)	< 0.001
	T4	6.173 (5.366-7.102)	< 0.001	2.510 (2.156-2.923)	< 0.001
	Unknown	2.465 (2.239-2.714)	< 0.001	1.471 (1.265-1.711)	< 0.001
N	N0	Reference		Reference	
	N1	3.255 (2.941-3.602)	< 0.001	1.210 (1.084-1.350)	< 0.001
	Unknown	1.428 (1.311-1.556)	< 0.001	1.176 (1.043-1.326)	0.008
M	M0	Reference		Reference	
	M1	4.035 (3.723-4.373)	< 0.001	1.781 (1.631-1.945)	< 0.001
	Unknown	0.946 (0.838-1.066)	0.361	0.823 (0.678-0.998)	0.047
Tumor Size	≤3 cm	Reference		Reference	
	3-5 cm	1.835 (1.694-1.989)	< 0.001	1.792 (1.651-1.946)	< 0.001
	≥5 cm	3.546 (3.304-3.806)	< 0.001	2.517 (2.300-2.754)	< 0.001
	Unknown	6.332 (5.748-6.975)	< 0.001	3.263 (2.886-3.688)	< 0.001
AFP	Negative	Reference		Reference	
	Positive	1.960 (1.826-2.105)	< 0.001	1.449 (1.347-1.559)	< 0.001
	Borderline	1.258 (0.627-2.522)	0.519	1.330 (0.662-2.674)	0.423
	Unknown	1.640 (1.479-1.819)	< 0.001	1.282 (1.151-1.428)	< 0.001
Radiation	None/unknown	Reference		Reference	
	Yes	1.249 (1.139-1.369)	< 0.001	0.653 (0.594-0.718)	< 0.001
Chemotherapy	None/unknown	Reference		Reference	
	Yes	0.744 (0.705-0.786)	< 0.001	0.466 (0.439-0.494)	< 0.001
Surgery	None	Reference		Reference	
	Tumor Destruction	0.317 (0.286-0.351)	< 0.001	0.388 (0.348-0.433)	< 0.001
	Surgical Resection	0.276 (0.246-0.310)	< 0.001	0.209 (0.183-0.240)	< 0.001
	Liver Transplantation	0.063 (0.049-0.082)	< 0.001	0.083 (0.064-0.108)	< 0.001
	Unknown	0.519 (0.247-1.090)	0.083	0.804 (0.382-1.691)	0.566
Fibrosis	Non-cirrhosis	Reference		Reference	
	Cirrhosis	1.259 (1.166-1.358)	< 0.001	1.166 (1.074-1.265)	< 0.001

^‡^, Black or African American; *, Includes American Indian/Alaska native, Asian, and Asian/Pacific Islander; ^#^, Includes widowed, never married, divorced, separated, unmarried, and domestic partner; T: tumour; N: node; M: metastasis; AFP: alpha fetoprotein; HR: hazard ratio; CI: confidence interval.

## Abbreviations

HCC: hepatocellular carcinoma
SEER: Surveillance, Epidemiology, and End Results
TNM: tumour, node, metastasis
AFP: alpha fetoprotein
AJCC: American Joint Committee on Cancer
HCSS: hepatocellular carcinoma-specific survival
OR: odds ratio
CI: confidence interval
